# A synthetic biological quantum optical system†
†Electronic supplementary information (ESI) available: Full details of all of the experimental materials and methods, together with detailed surface characterisation data obtained using X-ray photoelectron spectroscopy and spectroscopic ellipsometry. See DOI: 10.1039/c8nr02144a


**DOI:** 10.1039/c8nr02144a

**Published:** 2018-06-29

**Authors:** Anna Lishchuk, Goutham Kodali, Joshua A. Mancini, Matthew Broadbent, Brice Darroch, Olga A. Mass, Alexei Nabok, P. Leslie Dutton, C. Neil Hunter, Päivi Törmä, Graham J. Leggett

**Affiliations:** a Department of Chemistry , University of Sheffield , Brook Hill , Sheffield S3 7HF , UK . Email: Graham.Leggett@sheffield.ac.uk; b The Johnson Research Foundation and Department of Biochemistry and Biophysics , University of Pennsylvania , Philadelphia , PA 10104 , USA; c N. Carolina State University , Department of Chemistry , Raleigh , NC 27695 , USA; d Materials and Engineering Research Institute , Sheffield Hallam University , Howard St , Sheffield S1 1WB , UK; e Department of Molecular Biology and Biotechnology , University of Sheffield , Western Bank , Sheffield S10 2TN , UK; f COMP Centre of Excellence , Department of Applied Physics , Aalto University , School of Science , P.O. Box 15100 , 00076 Aalto , Finland

## Abstract

In strong plasmon–exciton coupling, a surface plasmon mode is coupled to an array of localized emitters to yield new hybrid light–matter states (plexcitons), whose properties may in principle be controlled *via* modification of the arrangement of emitters. We show that plasmon modes are strongly coupled to synthetic light-harvesting maquette proteins, and that the coupling can be controlled *via* alteration of the protein structure. For maquettes with a single chlorin binding site, the exciton energy (2.06 ± 0.07 eV) is close to the expected energy of the *Q*_*y*_ transition. However, for maquettes containing two chlorin binding sites that are collinear in the field direction, an exciton energy of 2.20 ± 0.01 eV is obtained, intermediate between the energies of the *Q*_*x*_ and *Q*_*y*_ transitions of the chlorin. This observation is attributed to strong coupling of the LSPR to an *H*-dimer state not observed under weak coupling.

## Abstract 2

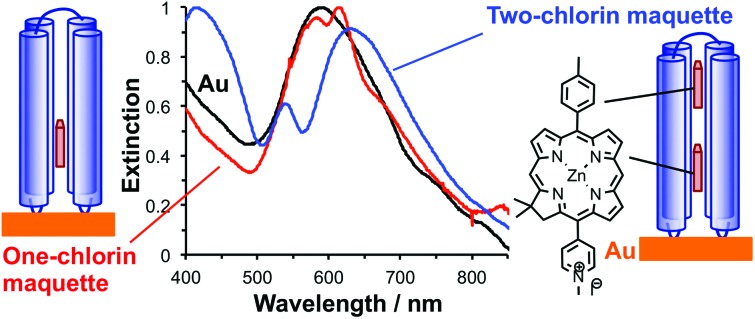
Strong coupling between plasmon modes and chlorins in synthetic light-harvesting maquette proteins yields hybrid light–matter states (plexcitons) whose energies are controlled by design of protein structure, enabling the creation of new states not seen under weak coupling.

## Introduction

Surface plasmons are collective excitations of surface electrons that may be coupled to incident electromagnetic radiation. Nanostructured noble metals give rise to strong plasmon absorptions, known as localized surface plasmon resonances (LSPRs), which may dominate their optical properties.^1^–^7^ Adsorption of biomolecules at the metal surface may lead to a shift in the LSPR energy, which can be exploited in biosensing applications.^7^,^8^ LSPRs may also couple to other metal nanoparticles, causing a shift in the position of the plasmon band in an extinction spectrum, or to optically active molecules,^7^–^12^ leading to an enhancement of the cross-sections for spectroscopic transitions^13^,^14^ Recently, however, there has been a great deal of interest in a very different kind of phenomenon: strong plasmon–exciton coupling, in which a delocalized electromagnetic mode supported at a metal surface is coupled with localized emitters^15^ to yield new hybrid states (“plexcitons”) *via* a linear combination of the plasmon and exciton states.^15^ These new states, above and below the energy of the plasmon mode, manifested as a splitting of the plasmon band in the extinction spectrum. Strong coupling has been reported in a variety of types of system, including ones based on dye molecules,^16^–^18^ *J*-aggregates^19^–^27^ and quantum dots.^15^

In strong plasmon–exciton coupling there is fast, coherent exchange of energy between the metal and the emitters.^15^,^28^ Importantly, it is a collective phenomenon – the LSPR couples to an array of emitters and the Rabi splitting energy depends on their concentration. An expected consequence of this is that spatially remote emitters may be coherent,^28^ leading to the possibility of exploiting strong plasmon–exciton coupling to achieve long-distance energy transfer or to create optical alloys by the coupling of different kinds of emitters. Such properties might have widespread applications, including quantum communications, quantum computing and solar energy capture. To explore these phenomena systematically, it would be valuable to have a means by which emitters could be organized in three dimensions within the plasmon mode. The present study examines the feasibility of using synthetic proteins to achieve this. Proteins are attractive for such fundamental studies because they have precisely defined structures that offer, in principle, control of both the density and orientation of binding sites for optically active ligands.

Photosynthetic antenna complexes capture sunlight with extraordinary efficiency, and funnel energy into reaction centres to drive the formation of charge-separated states.^29^ In purple bacteria and chloroplasts, the capture of photons leads to the formation of excitons that are delocalized across a number of pigment molecules within a complex *via* coherent electronic coupling;^30^–^34^ these excitons are then transferred between complexes in a sequence of Förster resonance energy transfer (FRET) steps before arriving at a reaction centre.^35^–^38^ These phenomena are important fundamentally for our understanding of photosynthesis, and there have been hopes that studies of biological light harvesting might also inform the design of synthetic photonic devices and materials.^38^ This has prompted intensive spectroscopic investigation of light harvesting complexes (LHCs).^14^,^32^,^33^,^39^–^41^ Central to this endeavour is the task of understanding how these molecules orchestrate sequences of energy transfer steps. However, the majority of published work has focused on naturally occurring molecules. This restricts the range of hypotheses that may be tested experimentally.

**Fig. 1:**
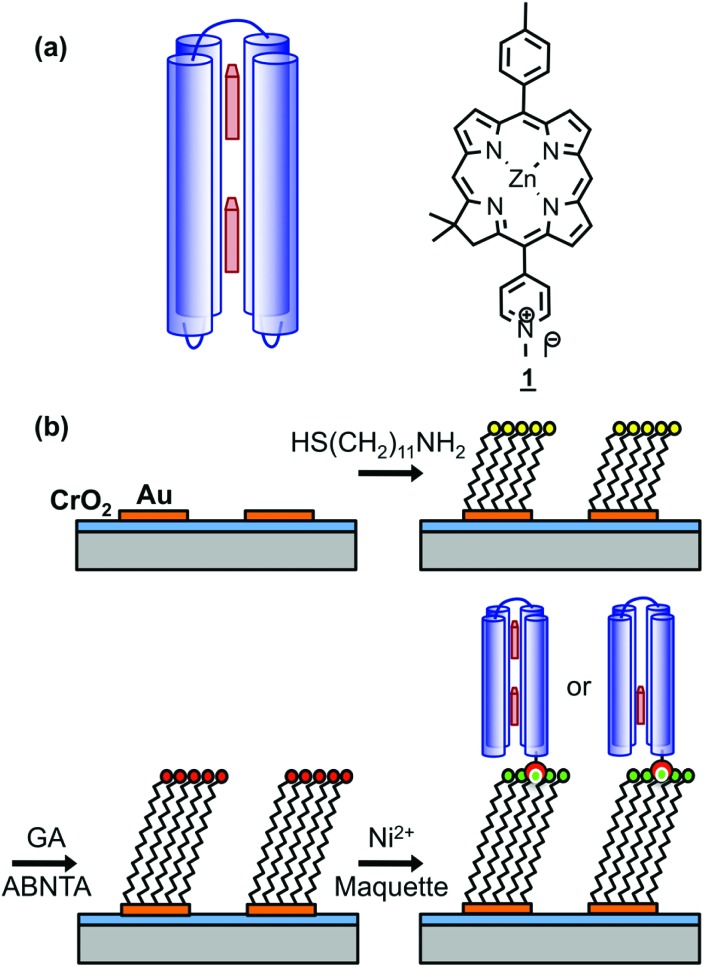
(a) Schematic diagrams showing the structure of BT6 maquettes, and the location of the two chlorins. (b) Schematic diagram showing the method used to site-specifically bind maquettes to gold nanostructures.

Genetic engineering of light-harvesting proteins has been used to explore a wider range of questions.^42^,^43^ Systematic variation of their pigment composition^44^ or the introduction of new functional units allow hypotheses about energy transfer to be tested.^45^ Alternatively measurements may be made on synthetic structures designed to replicate specific elements of photosynthetic mechanisms, for example DNA origami light-harvesting structures.^46^ To be able to address a broader range of fundamental questions, it would be useful to design *de novo* light-harvesting complexes in which, for example, the juxtaposition of pigment molecules could be controlled. Maquettes are synthetic proteins that consist of α-helical bundles that may be designed from scratch to incorporate cofactors that convey specific functions.^47^–^52^ They are designed from first principles, with minimal reference to natural protein sequences. They provide an ideal platform with which to address fundamental questions about the relationship between biological structure and function. The structures of a wide range of maquettes and their ligand binding sites have been determined by X-ray crystallography and other techniques, confirming that these *de novo* proteins can be designed with high precision.^53^ Recently the design of maquette light-harvesting complexes has been described.^54^ These proteins consist of four α-helices, in a single sequence, that self-assemble to form a bundle that encloses histidine binding sites that are able to bind to a variety of ligands including tetrapyrroles (Fig. 1a).^54^

Recently we described the strong coupling of LSPRs to excitons in light-harvesting proteins. We demonstrated that for LHCs from purple bacteria the plasmon–exciton system was well modelled using coupled harmonic oscillators, and was sensitive to changes in the pigment complement of the complexes.^55^ Here we describe a detailed investigation of strong coupling of LSPRs to two tetrahelical BT6 maquette proteins containing either one or two synthetic chlorins. The structures of these proteins may be selected *ab initio* to determine the precise presentation of pigment molecules within the plasmon mode, but their absorption spectra in solution are indistinguishable. We show that for BT6 maquettes containing two synthetic SE369 chlorins (**1**)^56^ placed in an approximately collinear arrangement in the field direction (Fig. 1a), the LSPR is hybridized with a state intermediate in energy between the chlorin *Q*_*x*_ and *Q*_*y*_ transitions. In contrast, the calculated exciton energy for a one-chlorin maquette is close to that of the chlorin *Q*_*y*_ transition. We propose that the data are best explained by coupling to a *H*-dimer state in the two-chlorin maquette, illustrating how protein structure may be designed in order to control the properties of the strongly coupled system and achieve coupling to states not observed under weak coupling.

## Results and discussion

### Attachment of maquettes to surfaces

Arrays of disc-shaped gold nanostructures covering cm^2^ regions were fabricated as described in detail previously.^57^Fig. 1b shows the procedure used to immobilize His_6_-tagged BT6 maquettes at the surfaces of the gold nanostructures. After adsorption of aminoundecanethiol (AUT), an aldehyde-terminated surface was produced by reaction with glutaraldehyde (GA), and then coupled to *N*-(5-amino-1-carboxypentyl) iminodiacetic acid to yield a nitrilotriacetic acid (NTA) functionalized film. This was complexed with Ni^2+^ to enable site-specific binding of His_6_-tagged BT6 maquettes. The surface modification process was investigated by using X-ray photoelectron spectroscopy (ESI†) to characterize model reactions carried out on continuous gold films (expected to have identical surface chemistry to the nanostructured materials), and to confirm the efficacy of the procedures used.

Ellipsometry was used to determine the efficacy of attachment of BT6 maquettes containing two SE369 chlorins (henceforth BT6-SE369_2_). These proteins are structurally identical to the one-chlorin maquettes, save for the presence of two internal chlorin binding sites rather than one, and both molecules coordinate to the surface *via* the same His-tag, so their surface attachment kinetics are expected to be identical. Table 1 shows the thicknesses determined after each stage of the process. After attachment of the maquette to the NTA-functionalized surface, an increase in thickness of 4.15 nm was observed. X-ray diffraction indicates that BT6 maquettes have dimensions of 4 nm (parallel to the α-helices) × 2 nm (perpendicular to the α-helices). The increase in thickness measured after attachment of maquettes to the surface is thus consistent with the formation of a close-packed layer in which their long axes are perpendicular to the surface. After measurement of the thickness of the maquette layer, the samples were treated with imidazole, which disrupts the interaction between the His tag and NTA/Ni^2+^. The fraction of maquettes removed from the surface was found to be ∼98%, confirming that attachment was predominantly *via* site-specific binding to the surface.

**Table 1:** Ellipsometric film thickness following successive surface modification steps (relative to clean gold)

Surface modification	Thickness/nm
Clean gold	—
+11-amino-1-undecanethiol	1.09 ± 0.14
+Glutaraldehyde	1.59 ± 0.11
+*N*-(5-amino-1-carboxypentyl) iminodiacetic acid	2.08 ± 0.10
+Maquette BT6 SE369	6.23 ± 0.25
After incubation with imidazole	2.13 ± 0.18

**Fig. 2:**
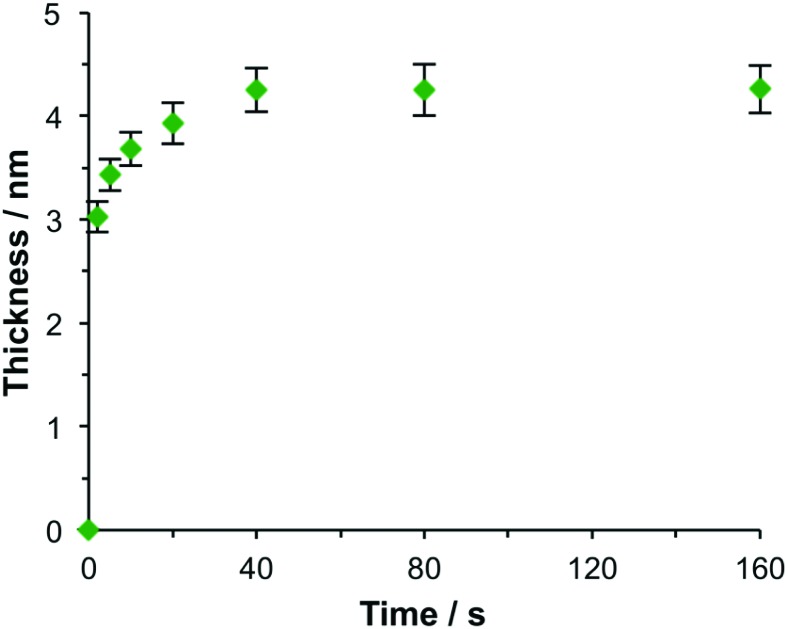
Ellipsometric thickness of the maquette film as a function of the time of immersion of NTA/Ni^2+^ functionalized gold films in a 500 nM solution of BT6-SE369_2_ in buffer.

The kinetics of maquette adsorption was studied using total internal reflection ellipsometry^58^ to measure the thickness of the adsorbed layer for unpatterned, polycrystalline gold films that had been immersed in a 500 nM solution of the maquette in buffer solution (Fig. 2). The surface chemistry of the polycrystalline films is expected to be indistinguishable from that of the gold nanostructures used to make plasmonic measurements, so they are a good model for the adsorption processes involved in the functionalization of gold nanostructures with maquettes. Ellipsometry is a well-established tool for the measurement of protein adsorption;^59^ the mean thickness is found to be proportional to the amount of adsorbed protein. The thickness increased rapidly at first, then more slowly, reaching a limiting value of ∼4.3 nm after a time of 40 min, consistent with the adsorption of maquettes to form a monolayer with a limiting coverage of *θ* = 1 in which the α-helices are aligned perpendicular to the gold surface. The behaviour was consistent with Langmuir-type adsorption, in which the surface coverage saturates at 1 monolayer, equivalent to ∼2.5 × 10^17^ m^–2^.

### Extinction spectra of two-chlorin maquettes

**Fig. 3:**
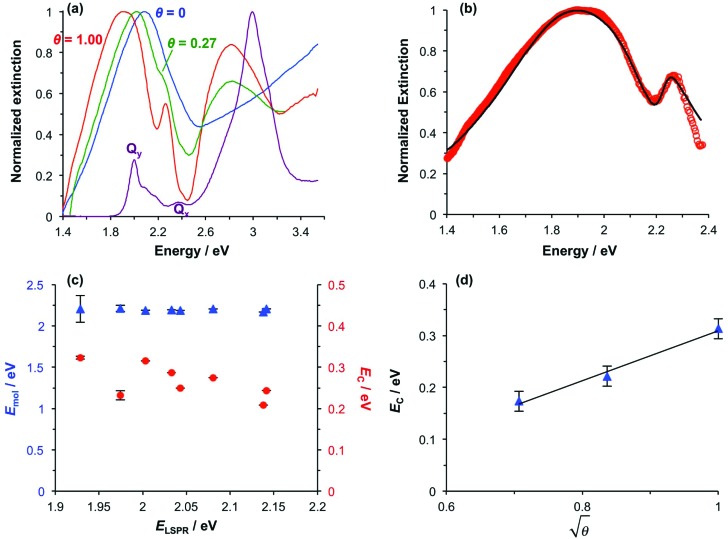
(a) Normalized extinction spectra of BT6-SE369_2_ maquettes in buffer solution (purple), clean gold nanostructures (blue) and gold nanostructures after attachment of BT6 maquettes with fractional coverages of 0.27 (green) and 1.00 (red). (b) Measured extinction spectrum (red symbols) and calculated spectrum obtained using the coupled harmonic oscillator model (black line) for a monolayer of BT6-SE369_2_. (c) Variation in the exciton energy (triangles) and scaled coupling energy (circles) as a function of the LSPR energy for a monolayer of BT6-SE369_2_ attached to gold nanostructures. (d) Variation in the scaled coupling energy as a function of the fractional surface coverage *θ* of BT6-SE369_2_.

Spectroscopic measurements were made using a spectrophotometer on samples of the two-chlorin maquette in solution and on arrays of gold nanostructures on glass slides to which the maquettes had been attached. In the solution-phase absorption spectrum of BT6-SE369_2_ (Fig. 3a, purple trace) the *Q*_*y*_ transition yields a peak at 620 nm (2.0 eV), with two smaller features at slightly higher energy due to vibronic coupling. The feature at 620 nm corresponds to the lowest energy (0,0) transition. The *Q*_*x*_ transition gives rise to a small feature at 522 nm (2.4 eV), and a strong Soret peak is observed at 414 nm (3.0 eV). These values are close to those reported previously by Aravindu *et al.* for SE369 in solution (615 and 407 nm, respectively, for the *Q*_*y*_ and Soret transitions, with a very weak feature at 516 nm due to the *Q*_*x*_ transition).^56^

Fig. 3a also shows an extinction spectrum of a clean array of gold nanostructures with height of 13 ± 1.5 nm and diameter 113 ± 23 nm at a pitch of 223 ± 13 nm (blue trace). A strong feature corresponding to the LSPR is observed at 2.04 eV (608 nm). After attachment of a monolayer of His_6_-tagged BT6-SE369_2_ maquettes to the array of gold nanostructures, the extinction spectrum is observed to change dramatically (Fig. 3a, red trace). The LSPR peak is split in an asymmetric fashion, to yield a large, broad feature at 1.88 eV (661 nm) and a smaller, narrower feature at 2.24 eV (553 nm). This type of splitting closely resembles that reported in our previous study of strong coupling of LSPRs to excitons in bacterial light-harvesting complexes,^55^ and is characteristic of an asymmetric Fano-type resonance between two oscillators with different linewidths, one broad (the LSPR) and one narrow (the maquette exciton).^60^ The two bands that result correspond to the two new states produced by hybridization of the LSPR and exciton states. The data in Fig. 3a thus indicate that the maquettes are strongly coupled to the LSPR associated with the gold nanostructures.

**Fig. 4:**
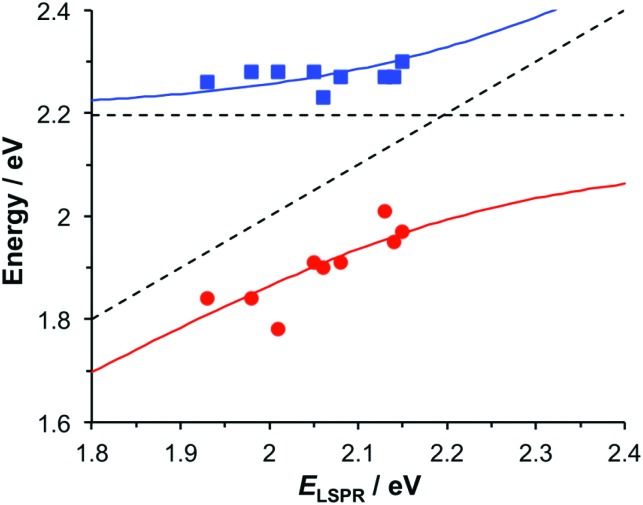
Dispersion curves for the plexcitonic states determined from experimental data (circles and squares) together with curves fitted using eqn (1).

To test this hypothesis, measurements were made for BT6-SE369_2_ maquettes coupled to a series of samples with varying LSPR energies *E*_LSPR_. The energies of the upper and lower polariton branches of the coupled system were determined from the spectra, and are plotted in Fig. 4. Dispersion curves were fitted using the relationship:^61^1
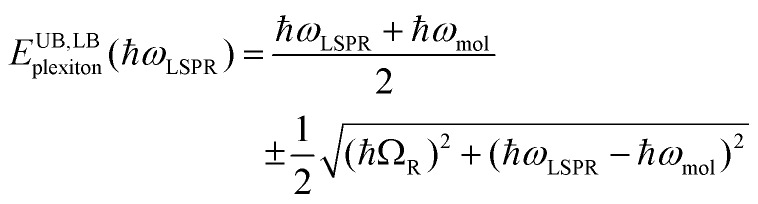
where *ħω*_LSPR_ and *ħω*_mol_ are the energies of the uncoupled LSPR and exciton, and *ħΩ*_R_ is the Rabi splitting, the separation between the upper (UB) and lower (LB) polariton branches at resonance (*ω*_LSPR_ = *ω*_mol_). From the fits to the data shown in Fig. 4, *ħΩ*_R_ was determined to be 0.31 eV. This allows us to test whether the systems studied here are in the strong coupling regime. A number of criteria have been used to define the threshold for strong plasmon–exciton coupling; these are order-of-magnitude criteria and may not always be met,^15^ for example when one of the coupled modes is much narrower than the other (as is the case here). One widely used measure is 
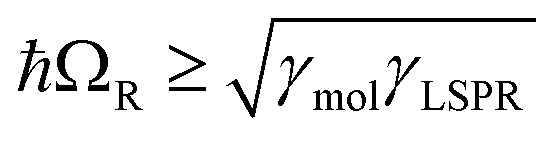
, where *γ*_LSPR_ and *γ*_mol_ are the linewidths of the uncoupled LSPR and exciton states. In the present case, *γ*_LSPR_ ∼ 0.6 eV and *γ*_mol_ ∼ 0.1 eV, hence the Rabi splitting should be greater than 0.24 eV, a condition that is satisfied here. Another criterion^15^ is that the Rabi splitting is greater than (*γ*_LSPR_ – *γ*_mol_)/2, which has the value 0.25 eV, again satisfied by the data in Fig. 4.

A strong feature is also observed at ∼2.8 eV (440 nm) in Fig. 3. The origin of this feature is not clear; its position appears to be independent of the nature of the protein, and similar features were observed in our previous study of bacterial LHCs.^55^ This 2.8 eV feature also lies at too high an energy to have been produced by plasmon–exciton coupling, so it appears to be unrelated to the splitting observed between 1.77 and 2.48 eV in the spectra in Fig. 3a.

### Modelling

The system was modelled as two coupled harmonic oscillators, using the methods described in our previous work for bacterial light harvesting complexes.^55^Fig. 3b shows an experimental spectrum (red symbols), together with a calculated spectrum (black line). A good fit was obtained. The data were examined further by obtaining the coupling constant for the system. In the coupled harmonic oscillator model, the coupling constant *g* has the dimensions of frequency squared. At resonance, the oscillators have the frequency *ω* and the splitting between the normal modes is ∼*g*/*ω*. When scaled to be expressed in units of energy, the coupling constant is *G* and the coupling energy (equal to the splitting between the normal modes) is *E*_C_ = *G*/*E*_LSPR_, where *E*_LSPR_ is the energy of the LSPR. Fig. 3c shows the variation in *E*_C_ with *E*_LSPR_ for seven different arrays of gold nanostructures (red circles). For each sample, the extinction spectrum was acquired after immobilization of BT6-SE369_2_ maquettes and fitted using the coupled harmonic oscillator model. Good fits were obtained for all of the samples. The mean coupling energy determined from the fitting was 0.27 ± 0.04 eV, close to the value determined from Fig. 4.

In the strong coupling regime a surface plasmon mode is coupled to an array of emitters; the density of dipoles in this array should thus influence the coupling energy. According to the microscopic theory,^15^,^17^,^62^
2
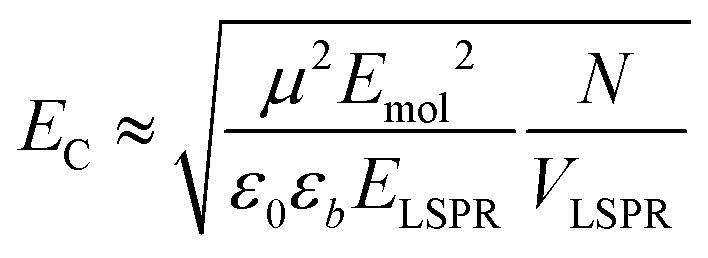
where *μ* is the transition dipole moment associated with each of the *N* dipoles within the LSPR mode volume VLSPR, *ε*_0_ is the permittivity of free space, *ε*_*b*_ is the relative permittivity of the background medium (in our case one, the medium is air, with only an ultrathin protein layer). Hence the coupling energy should vary with the square root of the density of dipoles within the plasmon mode volume, which we estimated previously to extend up to 35 nm from the gold surface.^55^

For a layer of adsorbed maquettes, the density of transition dipole moments is determined by the fractional coverage *θ*, where *θ* = 1 corresponds to monolayer coverage. Thus the coupling energy is expected to vary as a function of 
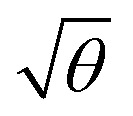
. The adsorption curve for BT6-SE369_2_ (ESI†) was used to prepare arrays of nanostructures with variable fractional coverages of protein. Arrays of nanostructures were prepared, derivatized with NTA/Ni^2+^ and immersed in a solution of maquettes in buffer for varying periods of time to achieve controlled differences in fractional coverage. Fig. 3a shows qualitatively that the coupling varied with fractional coverage. Three extinction spectra were all acquired for the same array of gold nanostructures. After deposition of the monolayer sample (red trace), the sample was cleaned using piranha solution to remove all molecular adsorbates. The clean array was characterized to ensure that the extinction spectrum was unchanged, then functionalized to yield an NTA/Ni^2+^ termination. The green trace in Fig. 3a was then acquired at a fractional coverage of 0.27. At this partial coverage, plasmon–exciton coupling occurs, but the system is not strictly in the strong coupling regime. The upper polariton branch appears as a shoulder on the lower polariton branch.

Spectra were fitted using the coupled harmonic oscillator model to yield the coupling constant for the system. The scaled coupling energy, *E*_C_ = *G*/*E*_LSPR_, is shown as a function of the square root of the fractional coverage in Fig. 3d. It is clear that the relationship between the coupling energy and *θ* is linear, as expected from eqn (2).

It is important to note that piranha solution is a very strong oxidizing agent, and when it is used for repeated cleaning of nanostructures, they can undergo degradation. Extensive repetition of measurements using the same sample is thus difficult and error bars cannot be fitted to the data. However, repetition of the experiments described above using a different sample yielded extinction spectra that exhibited similar qualitative changes, and when modelled, these spectra also yielded a linear relationship between *E*_C_ and 
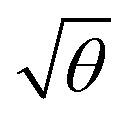
. It is significant that while the coupling energy yielded the expected dependence upon the density of dipoles, the exciton energy *E*_mol_ for these experiments yielded a mean value of 2.20 ± 0.01 eV, in exact agreement with the results presented in Fig. 3b.

### Chlorin coupling

Fig. 3c also shows the exciton energy BT6-SE369_2_ as a function of *E*_mol_, also obtained from fitting the spectra (blue triangles). As expected, *E*_mol_ remains constant as *E*_LSPR_ is varied. The mean value of *E*_mol_ was 2.20 ± 0.01 eV, intermediate between the energies of the *Q*_*y*_ (2.0 eV) and *Q*_*x*_ (2.4 eV) transitions. In our previous study of bacterial light-harvesting complexes, the values for the exciton energies determined using the coupled harmonic oscillator model were very close to the values of known transitions, either the carotenoid S2 transition or the bacteriochlorophyll *Q*_*x*_ transition. The value obtained for *E*_mol_ for BT6-SE369_2_ using the same model is not equal to the energy of either the *Q*_*y*_ or the *Q*_*x*_ transition.

**Fig. 5:**
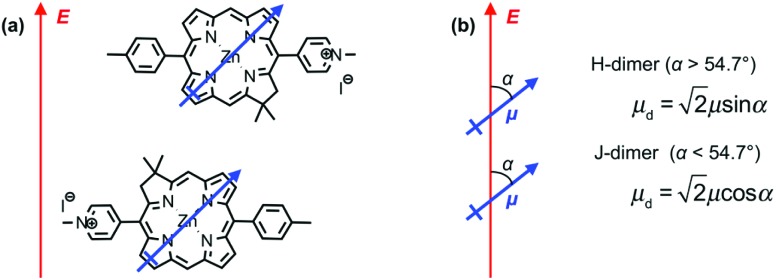
(a) Possible alignment of pairs of SE369 chlorins in maquettes. The blue arrow represents the *Q*_*y*_ transition dipole moment, and the red arrow the direction of the field associated with the surface plasmon mode. (b) Possible coupling schemes for chlorin dimers.

The most reasonable explanation is that the transition dipole moments of the chlorins in BT6-SE369_2_ are coupled. Although the *Q*_*y*_ transition is very much more intense than the *Q*_*x*_ transition in SE369 (Fig. 3a, purple trace), and the *Q*_*x*_ transition is significantly further in energy from the LSPR than the *Q*_*y*_ transition, it is not possible to determine *a priori* whether the coupled state corresponding corresponds to a type of *J*-dimer (red shifted coupled *Q*_*x*_ transitions) or to a type of *H*-dimer (blue-shifted coupled *Q*_*y*_ transitions)^63^ in the strongly coupled plasmon–exciton system. Such conventional coupling schemes may represent a rather simplistic approximation to the type of coupling in our system consisting of a large number of emitters and strong plasmonic fields. However, they provide a means to explore the coupling quantitatively. SE369 is an amphiphilic chlorin, with a polar pyridinium salt conferring hydrophilicity at one end of the molecule. The chlorin coordinates to the His site *via* the tetrapyrrole ring, while the pyridinium end of the molecule is located near to the hydrophilic outer surface of the protein. Fig. 5 shows a possible alignment of the chlorins and the orientation of the *Q*_*y*_ transition dipole moment. It is clear that a small change in orientation of the chlorins could lead to the possibility of either *J*- or *H*-coupling for either the *Q*_*y*_ or the *Q*_*x*_ transition.

Coupling of transition dipole moments in tetrapyrroles has been widely reported. For example, Shoji *et al.* report a red shift equivalent to ∼0.23 eV in π-stacked assemblies of bacteriochlorophylls,^64^ while Furumaki *et al.* reported a shift of ∼0.17 eV.^65^ The shift observed here (∼0.2 eV) is similar. However, the separation between the chlorins in BT6-SE369_2_ is ∼2 nm. At this distance, dipole coupling is expected to be weaker, and the solution-phase absorption spectrum shows no evidence of such coupling (*i.e.* the spectrum is similar to that observed for the chlorin alone). In solution the excitation is incoherent and the interaction is not expected to be strong at the relatively large separation of 2 nm. In a recent study, Kodali *et al.* described incoherent coupling in maquettes that incorporate different pigments, demonstrating that dipole coupling is possible in these molecules, albeit weakly in the solution phase.^54^ However, in strong plasmon–exciton coupling, there is coherent energy exchange between the surface plasmon and the emitters.^15^,^28^,^66^ It is possible that coupled emitter states that are not observed under weak coupling become visible when strongly coupled to the plasmonic resonance.

### Comparative investigation of a one-chlorin maquette

**Fig. 6:**
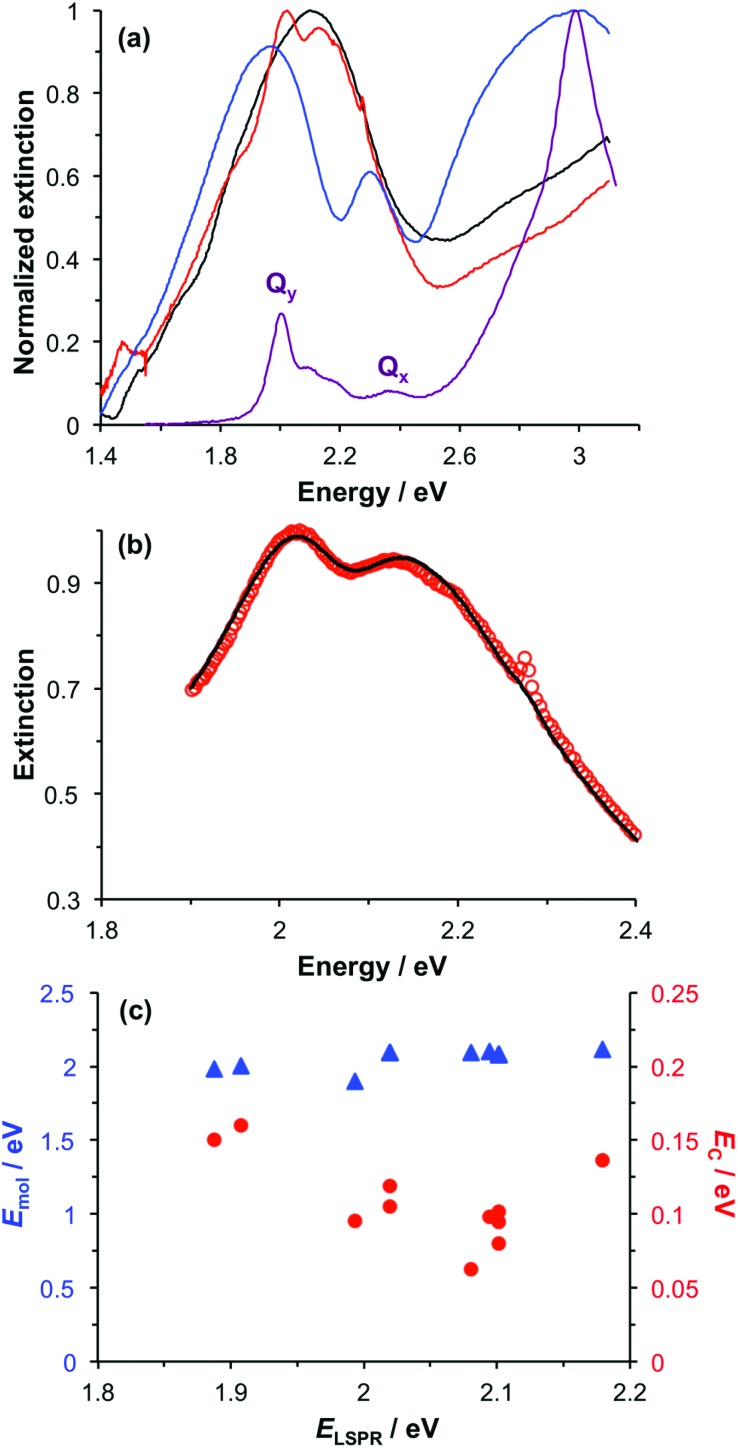
(a) Extinction spectra of BT6-SE369_1_ maquettes in buffer solution (purple) and of an array of gold nanostructures before (black) and after attachment of monolayers of BT6-SE369_1_ (red) and BT6-SE369_2_ (blue). (b) Measured extinction spectrum (red symbols) and calculated spectrum obtained using the coupled harmonic oscillator model (black line) for a monolayer of BT6-SE369_1_. (c) Variation in the exciton energy (triangles) and scaled coupling energy (circles) as a function of the LSPR energy for a monolayer of BT6-SE369_1_ attached to gold nanostructures.

To test the hypothesis that transition dipole moments are coupled in BT6-SE369_2_, measurements were made using a variant of BT6 containing only a single chlorin binding site (BT6-SE369_1_). The solution-phase spectrum of this maquette (Fig. 6a, purple trace) is indistinguishable from that of BT6-SE369_2_. Monolayers of the His-tagged protein were formed by attachment to NTA/Ni^2+^ terminated SAMs. Ellipsometry confirmed that, as expected, the kinetics of adsorption were similar to those of BT6-SE369_2_. A thickness of ∼4.5 nm was measured for a protein monolayer attached to a polycrystalline gold film, close to the value measured for BT6-SE369_2_, confirming that in both cases the protein was oriented with its long axis perpendicular to the metal surface.

Fig. 6a shows an extinction spectrum of an array of gold nanostructures before (black) and after attachment of BT6-SE369_1_ (red) and BT6-SE369_2_ (blue). It is immediately clear that the separation between the upper and lower polariton branches is reduced for the single-chlorin maquette. This indicates a substantial difference in the plasmon–exciton coupling for these two proteins, which have indistinguishable absorption spectra in solution. The extinction spectrum for the sample derivatized with the one-chlorin maquette was fitted using our coupled harmonic oscillator model (Fig. 6b). A good fit was obtained. The coupling energy was found to be reduced. For a group of 11 different arrays, a mean coupling energy of 0.11 eV was obtained, confirming that for the one-chlorin maquette, in contrast to the two-chlorin maquette, the system approaches the strong coupling regime but may not be said to be strongly coupled.

Measurements were made on a series of different arrays after attachment of BT6-SE369_1_. The spectra were modelled, and the exciton energies and coupling energies were determined (Fig. 6c). The mean value of the exciton energy for these samples was 2.06 ± 0.07 eV, significantly different from the value obtained for the two-chlorin maquette, and close to the energy of the *Q*_*y*_ transition. This supports the hypothesis that the larger exciton energy calculated for BT6-SE369_2_ was due to the formation of a coupled state in that molecule.

From the microscopic theory of strong coupling it is known that 
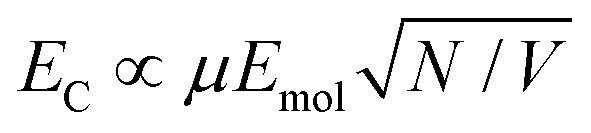
. The values of *E*_C_ and the exciton energy *E*_mol_ are obtained from the fitting of extinction spectra. If the LSPR couples to individual chlorin dipoles in a monolayer of one-chlorin maquettes, and to chlorin dimers in a monolayer of two-chlorin maquettes, then 
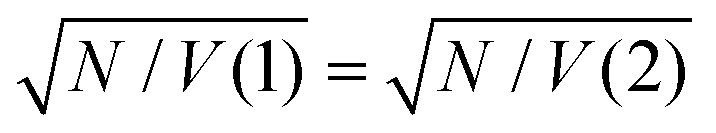
 and we can write the ratio of the dipole moments in the two maquettes as:3
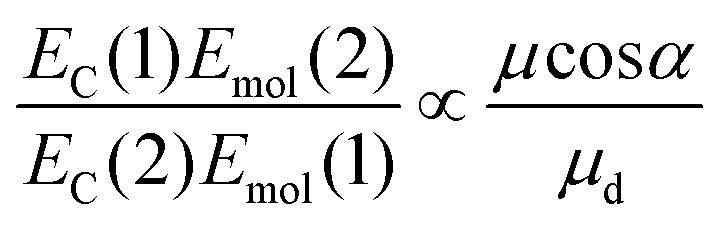
where *μ* is the transition dipole moment of a single chlorin, *α* is the angle between *μ* and the field direction and *μ*_d_ is the dipole moment of the chlorin dimer in the two-chlorin maquette.

For a *J*-dimer (*α* < 54.7°), 
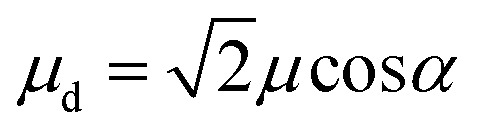
 (Fig. 5) hence:4
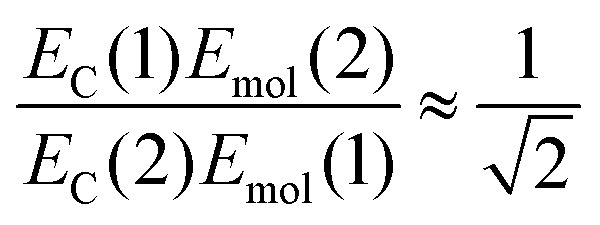



The mean values of the coupling energies were 0.11 ± 0.01 eV and 0.27 ± 0.04 eV for the one- and two-chlorin maquettes; combining these with the calculated exciton energies, we obtain a value for the dipole ratio of 0.045 ± 0.11, significantly different from the value expected from eqn (3).

If instead the coupled chlorins are modelled as an *H*-dimer, the dipole ratio is given by:5
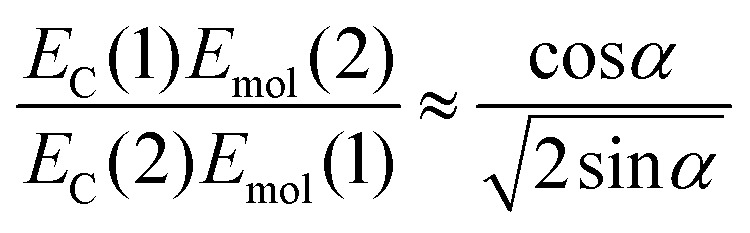



For eqn (5) the dipole ratio approaches the experimental value for *α* ∼ 58°, consistent with the formation of an *H*-dimer. Thus we conclude that in the two-chlorin maquette the LSPR couples to a *Q*_*y*_ dimer state.

At *α* ∼ 58°, close to the angle that separates *H*- and *J*-dimer formation, conventional dipole coupling would yield a very small coupling energy, consistent with the absence of evidence for a dimer state in the solution phase absorption spectrum. However, non-local couplings between emitters have been postulated in strong plasmon–exciton coupling,^28^,^66^ as a result of ultra-fast exchange of energy between an array of dipoles and the plasmon mode. Such interactions may explain the substantial blue shift observed here for the two-chlorin maquette.

The increased exciton energy in the two-chlorin maquette is insufficient on its own to explain the very large increase in the coupling energy. However, *H*-dimer formation would be expected to yield a substantial increase in the transition dipole moment (we estimate by a factor of *ca.* 2). Thus coupling between the chlorins in the strongly coupled system could increase both *μ* and *E*_mol_ in eqn (2), leading to the substantial increase in *E*_C_ observed in Fig. 3c.

It is important to note that the use of an *H*-dimer model here may represent a significant over-simplification of what may be a more complex coupling mechanism. Given that in strong plasmon–exciton coupling the plasmon mode couples to an array of emitters, it is indeed plausible that the observed couplings involve chlorins in different proteins. Undoubtedly the geometry of the two-chlorin maquette – with two collinear pigment molecules – has a decisive influence. But it may be an over-simplification to assume that discrete *H*-dimers are formed in the strongly coupled system. A full understanding of the coupling modality requires further theoretical and experimental work, which is beyond the scope of the present study.

**Fig. 7:**
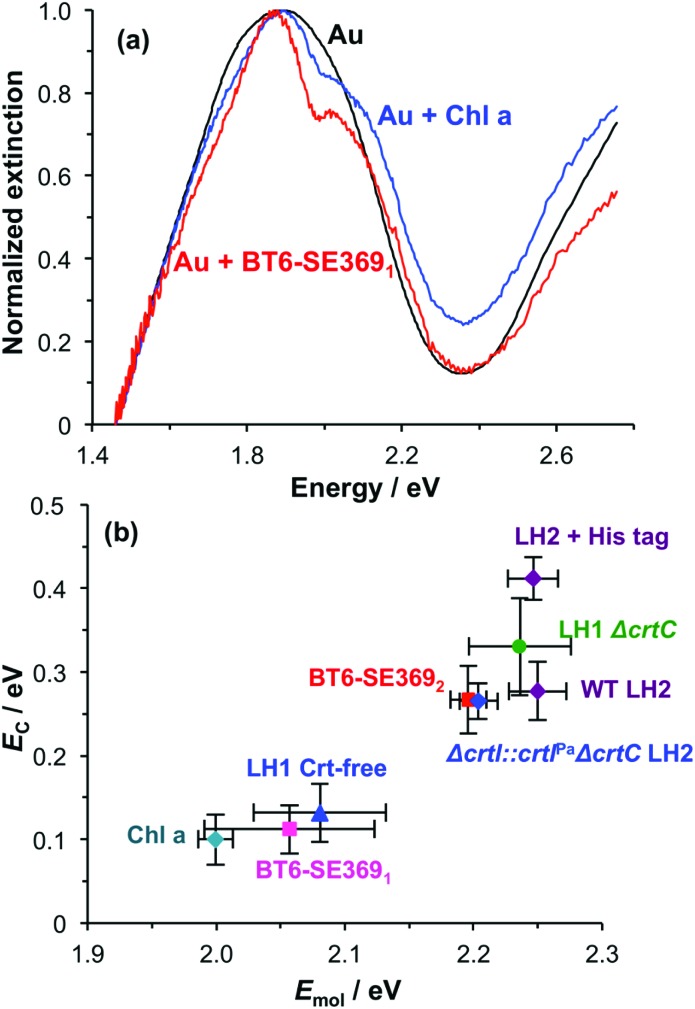
(a) Extinction spectra for clean array of gold nanostructures before (black) and after attachment of BT6-SE369_1_ (red) and chlorophyll *a* (blue). (b) Plot of mean coupling energy *E*_C_ as a function of the exciton energy *E*_mol_ for a variety of light-harvesting complexes and for chlorophyll *a*.

As a final control, chlorophyll *a* (Chl *a*) was extracted from spinach and treated with glacial acetic acid to yield an ester linker which was first hydrolysed to a carboxylic acid, and then converted to an acyl chloride for attachment to a self-assembled monolayer of aminoundecanethiol (the second stage of the process represented in Fig. 1b). The principal absorption maximum of Chl *a* in the red region of the spectrum occurs at 1.86 eV (665 nm), close to that for BT6-SE369_1_. Extinction spectra were acquired for an array of gold nanostructures after chlorophyll *a* had been attached, and then subsequently after cleaning the array in piranha solution and attachment of BT6-SE369_1_. It is striking that the spectra (Fig. 7a) are very similar. As expected, the splitting of the plasmon band is very similar. These data further emphasize the unexpected character of the behaviour of the two-chlorin maquette BT6-SE369_2_, and lend further support to the hypothesis that the LSPR couples to a *Q*_*y*_ dimer state in the two-chlorin maquette.

The significance of this effect is further illustrated in Fig. 7b which shows mean coupling energies as a function of the exciton energy for a selection of LHCs studied here and in a previous paper.^55^ It is striking that in Fig. 7b, BT6-SE369_2_ stands apart from the chlorin-only complexes BT6-SE369_1_ and blue LH1 (a mutant of LH1 from *R. sphaeroides* that contains no carotenoids). Instead, it lies closer to the carotenoid-containing light-harvesting complexes, LH2 and LH1 Δ*crtC*. Although dimerization of the pigment molecules in BT6-SE369_2_ increases the coupling energy, it is significant that eqn (4) suggests that at an angle of orientation relative to the field direction of 58° an *H*-dimer will have a transition dipole moment *ca.* 2 times the value obtained for a single chlorin. This large enhancement of the dipole moment probably accounts for the large splitting energy and qualitatively very different extinction spectrum for BT6-SE369_2_ relative to those for BT6-SE369_1_.

The fact that the solution-phase absorption spectra of these two proteins are indistinguishable suggests that coupling of the LSPR to the *H*-dimer state in BT6-SE369_2_ is specifically a quantum optical phenomenon: for these small dipoles at a separation of 2 nm, conventional dipole coupling does not lead to sufficiently effective energy exchange for an *H*-dimer state to be observed. However, in strong coupling, ultra-fast energy exchange occurs *via* the plasmon mode and enables the chlorins to couple to form an *H*-dimer. This illustrates the potential of strong plasmon–exciton coupling to create new types of optical phenomena. The fact that the coupling can be manipulated by alteration of the protein structure (in particular the change from two to one chlorin binding sites) illustrates the capability that synthetic biology presents for exploring strong light–matter interactions, because of the way that protein structures can be engineered to control the presentation of optically active molecules in 3D. This approach may provide a template for a new kind of synthetic biological metamaterial, in which *ab initio* biomolecular design is used to produce materials designed to exhibit specific optical behaviour.

## Conclusions

Histidine-labelled tetrapeptide maquette light-harvesting complexes containing synthetic chlorins may be bound site-specifically to arrays of gold nanostructures functionalized with NTA-terminated monolayers. A pronounced splitting of the plasmon band is observed in extinction spectra of these systems is consistent with an asymmetric Fano-type resonance. These resonances are attributed to strong coupling between the LSPR and excitons in the maquettes. The couplings are effectively modelled as coupled harmonic oscillators. The coupling energy *E*_C_ is found to be proportional to the square root of the surface coverage, consistent with microscopic theory of strong coupling. For maquettes containing only a single chlorin, an exciton energy of 2.06 ± 0.07 eV is determined, close to the energy of the *Q*_*y*_ transition and close to the energies obtained for chlorophyll *a* and for bacterial LHCs containing no carotenoids. The exciton energy was found to be 2.20 ± 0.01 eV for maquettes containing two chlorins, however, intermediate between the energies of the *Q*_*x*_ and *Q*_*y*_ transitions. A peak corresponding to this energy is not seen in the absorption spectrum of the maquettes. It is attributed to the formation of a *H*-dimer as a result of fast coherent energy exchange between the pigment molecules in the strongly coupled regime. These data illustrate the potential that is offered by strong coupling in conjunction with synthetic biology, by facilitating the design of hybrid materials in which the arrangement of emitters within the plasmon mode volume is organized precisely, enabling the creation of bespoke optical states.

## Conflicts of interest

There are no conflicts to declare.

## Supplementary Material

Supplementary information

## References

[cit1] Ebbesen T. W., Lezec H. J., Ghaemi H. F., Thio T., Wolff P. A. (1998). Nature.

[cit2] Maier S. A., Brongersma M. L., Kik P. G., Meltzer S., Requicha A. A. G., Atwater H. A. (2001). Adv. Mater..

[cit3] Barnes W. L., Dereux A., Ebbesen T. W. (2003). Nature.

[cit4] Ergin T., Stenger N., Brenner P., Pendry J. B., Wegener M. (2010). Science.

[cit5] Fang N., Lee H., Sun C., Zhang X. (2005). Science.

[cit6] Hibbins A. P., Evans B. R., Sambles J. R. (2005). Science.

[cit7] Anker J. N., Hall W. P., Lyandres O., Shah N. C., Zhao J., Van Duyne R. P. (2008). Nat. Mater..

[cit8] Rosi N. L., Mirkin C. A. (2005). Chem. Rev..

[cit9] Nieder J. B., Bittl R., Brecht M. (2010). Angew. Chem., Int. Ed..

[cit10] Bujak Å., Czechowski N., Piatkowski D., Litvin R., Mackowski S., Brotosudarmo T. H. P., Cogdell R. J., Pichler S., Heiss W. (2011). Appl. Phys. Lett..

[cit11] Brecht M., Hussels M., Nieder J. B., Fang H., Elsässer C. (2012). Chem. Phys..

[cit12] Bujak L., Olejnik M., Brotosudarmo T. H. P., Schmidt M. K., Czechowski N., Piatkowski D., Aizpurua J., Cogdell R. J., Heiss W., Mackowski S. (2014). Phys. Chem. Chem. Phys..

[cit13] Brinks D., Castro-Lopez M., Hildner R., van Hulst N. F. (2013). Proc. Natl. Acad. Sci. U. S. A..

[cit14] Wientjes E., Renger J., Curto A. G., Cogdell R., van Hulst N. F. (2014). Nat. Commun..

[cit15] Törmä P., Barnes W. L. (2015). Rep. Prog. Phys..

[cit16] Hakala T. K., Toppari J. J., Kuzyk A., Pettersson M., Tikkanen H., Kunttu H., Törmä P. (2009). Phys. Rev. Lett..

[cit17] Shi L., Hakala T. K., Rekola H. T., Martikainen J. P., Moerland R. J., Törmä P. (2014). Phys. Rev. Lett..

[cit18] Väkeväinen A. I., Moerland R. J., Rekola H. T., Eskelinen A. P., Martikainen J. P., Kim D. H., Törmä P. (2014). Nano Lett..

[cit19] Bellessa J., Bonnand C., Plenet J. C., Mugnier J. (2004). Phys. Rev. Lett..

[cit20] Dintinger J., Klein S., Bustos F., Barnes W. L., Ebbesen T. W. (2005). Phys. Rev. B: Condens. Matter Mater. Phys..

[cit21] Sugawara Y., Kelf T. A., Baumberg J. J., Abdelsalam M. E., Bartlett P. N. (2006). Phys. Rev. Lett..

[cit22] Wurtz G. A., Evans P. R., Hendren W., Atkinson R., Dickson W., Pollard R. J., Zayats A. V., Harrison W., Bower C. (2007). Nano Lett..

[cit23] Fofang N. T., Park T.-H., Neumann O., Mirin N. A., Nordlander P., Halas N. J. (2008). Nano Lett..

[cit24] Vasa P., Wang W., Pomraenke R., Lammers M., Maiuri M., Manzoni C., Cerullo G., Lienau C. (2013). Nat. Photonics.

[cit25] Zengin G., Wersäll M., Nilsson S., Antosiewicz T. J., Käll M., Shegai T. (2015). Phys. Rev. Lett..

[cit26] Fales A. M., Norton S. J., Crawford B. M., DeLacy B. G., Vo-Dinh T. (2015). Phys. Chem. Chem. Phys..

[cit27] DeLacy B. G., Miller O. D., Hsu C. W., Zander Z., Lacey S., Yagloski R., Fountain A. W., Valdes E., Anquillare E., Soljačić M., Johnson S. G., Joannopoulos J. D. (2015). Nano Lett..

[cit28] Aberra Guebrou S., Symonds C., Homeyer E., Plenet J. C., Gartstein Y. N., Agranovich V. M., Bellessa J. (2012). Phys. Rev. Lett..

[cit29] Scholes G. D., Fleming G. R., Olaya-Castro A., van Grondelle R. (2011). Nat. Chem..

[cit30] Cory M. G., Zerner M. C., Hu X., Schulten K. (1998). J. Phys. Chem. B.

[cit31] Damjanović A., Ritz T., Schulten K. (1999). Phys. Rev. E: Stat. Phys., Plasmas, Fluids, Relat. Interdiscip. Top..

[cit32] Lee H., Cheng Y.-C., Fleming G. R. (2007). Science.

[cit33] Engel G. S., Calhoun T. R., Read E. L., Ahn T.-K., Mancal T., Cheng Y.-C., Blankenship R. E., Fleming G. R. (2007). Nature.

[cit34] Chenu A., Scholes G. D. (2015). Annu. Rev. Phys. Chem..

[cit35] Şener M., Strümpfer J., Hsin J., Chandler D., Scheuring S., Hunter C. N., Schulten K. (2011). ChemPhysChem.

[cit36] Strümpfer J., Şener M., Schulten K. (2012). J. Phys. Chem. Lett..

[cit37] Fassioli F., Dinshaw R., Arpin P. C., Scholes G. D. (2014). J. R. Soc., Interface.

[cit38] Bredas J.-L., Sargent E. H., Scholes G. D. (2017). Nat. Mater..

[cit39] Ostroumov E. E., Mulvaney R. M., Cogdell R. J., Scholes G. D. (2013). Science.

[cit40] Arpin P. C., Turner D.D.
B.B., McClure S. D., Jumper C. C., Mirkovic T., Challa J. R., Lee J., Teng C. Y., Green B. R., Wilk K. E., Curmi P. M. G., Hoef-Emden K., McCamant D. W., Scholes G. D. (2015). J. Phys. Chem. B.

[cit41] Hildner R., Brinks D., Nieder J. B., Cogdell R. J., van Hulst N. F. (2013). Science.

[cit42] Herek J. L., Fraser N. J., Pullerits T., Martinsson P., Polívka T., Scheer H., Cogdell R. J., Sundström V. (2000). Biophys. J..

[cit43] Chi S. C., Mothersole D. J., Dilbeck P., Niedzwiedzki D. M., Zhang H., Qian P., Vasilev C., Grayson K. J., Jackson P. J., Martin E. C., Li Y., Holten D., Neil Hunter C. (2015). Biochim. Biophys. Acta, Bioenerg..

[cit44] Reddy K. R., Jiang J., Krayer M., Harris M. A., Springer J. W., Yang E., Jiao J., Niedzwiedzki D. M., Pandithavidana D., Parkes-Loach P. S., Kirmaier C., Loach P. A., Bocian D. F., Holten D., Lindsey J. S. (2013). Chem. Sci..

[cit45] Grayson K. J., Faries K. M., Huang X., Qian P., Dilbeck P., Martin E. C., Hitchcock A., Vasilev C., Yuen J. M., Niedzwiedzki D. M., Leggett G. J., Holten D., Kirmaier C., Neil Hunter C. (2017). Nat. Commun..

[cit46] Dutta P. K., Levenberg S., Loskutov A., Jun D., Saer R., Beatty J. T., Lin S., Liu Y., Woodbury N. W., Yan H. (2014). J. Am. Chem. Soc..

[cit47] Robertson D. E., Farid R. S., Moser C. C., Urbauer J. L., Mulholland S. E., Pidikiti R., Lear J. D., Wand A. J., DeGrado W. F., Dutton P. L. (1994). Nature.

[cit48] Ye S., Discher B. M., Strzalka J., Xu T., Wu S. P., Noy D., Kuzmenko I., Gog T., Therien M. J., Dutton P. L., Blasie J. K. (2005). Nano Lett..

[cit49] Lichtenstein B. R., Farid T. A., Kodali G., Solomon L. A., Anderson J. L. R., Sheehan M. M., Ennist N. M., Fry B. A., Chobot S. E., Bialas C., Mancini J. A., Armstrong C. T., Zhao Z., Esipova T. V., Snell D., Vinogradov S. A., Discher B. M., Moser C. C., Dutton P. L. (2012). Biochem. Soc. Trans..

[cit50] Koder R. L., Anderson J. L. R., Solomon L. A., Reddy K. S., Moser C. C., Dutton P. L. (2009). Nature.

[cit51] Farid T. A., Kodali G., Solomon L. A., Lichtenstein B.B.
R.R., Sheehan M. M., Fry B. A., Bialas C., Ennist N. M., Siedlecki J. A., Zhao Z., Stetz M. A., Valentine K. G., Anderson J. L. R., Wand A. J., Discher B. M., Moser C. C., Dutton P. L. (2013). Nat. Chem. Biol..

[cit52] Solomon L. A., Kodali G., Moser C. C., Dutton P. L. (2014). J. Am. Chem. Soc..

[cit53] Moser C. C., Ennist N. M., Mancini J. A. and Dutton P. L., in Mechanisms of Primary Energy Transduction in Biology, The Royal Society of Chemistry, 2018, pp. 1–24.

[cit54] Kodali G., Mancini J. A., Solomon L. A., Episova T. V., Roach N., Hobbs C. J., Wagner P., Mass O. A., Aravindu K., Barnsley J. E., Gordon K. C., Officer D. L., Dutton P. L., Moser C. C. (2017). Chem. Sci..

[cit55] Tsargorodska A., Cartron M. L., Vasilev C., Kodali G., Mass O. A., Baumberg J. J., Dutton P. L., Hunter C. N., Törmä P., Leggett G. J. (2016). Nano Lett..

[cit56] Aravindu K., Mass O., Vairaprakash P., Springer J. W., Yang E., Niedzwiedzki D. M., Kirmaier C., Bocian D. F., Holten D., Lindsey J. S. (2013). Chem. Sci..

[cit57] Tsargorodska A., El Zubir O., Darroch B., Cartron M. l. L., Basova T., Hunter C. N., Nabok A. V., Leggett G. J. (2014). ACS Nano.

[cit58] Arwin H., Poksinski M., Johansen K. (2004). Appl. Opt..

[cit59] Rabe M., Verdes D., Seeger S. (2011). Adv. Colloid Interface Sci..

[cit60] Gallinet B., Martin O. J. F. (2011). Phys. Rev. B: Condens. Matter Mater. Phys..

[cit61] Schlather A. E., Large N., Urban A. S., Nordlander P., Halas N. J. (2013). Nano Lett..

[cit62] Agranovich V. M., Litinskaia M., Lidzey D. G. (2003). Phys. Rev. B: Condens. Matter Mater. Phys..

[cit63] Heyne B. (2016). Photochem. Photobiol. Sci..

[cit64] Shoji S., Ogawa T., Hashishin T., Ogasawara S., Watanabe H., Usami H., Tamiaki H. (2016). Nano Lett..

[cit65] Furumaki S., Vacha F., Hirata S., Vacha M. (2014). ACS Nano.

[cit66] Galego J., Garcia-Vidal F. J., Feist J. (2017). Phys. Rev. Lett..

