# Supplementary material for: A synthetic biological quantum optical system
Source: Nanoscale. 2018 Jun 29;10(27):13064–73. doi: 10.1039/c8nr02144a (PMC6044288; doi:10.1039/c8nr02144a)
Supplement: Supplementary file 1 [file NR-010-C8NR02144A-s001.pdf]

Electronic Supplementary Material (ESI) for Nanoscale.
This journal is © The Royal Society of Chemistry 2018

Supporting Information

A Synthetic Biological Quantum Optical System

Anna Lishchuk,[1] Goutham Kodali,[2] Joshua A. Mancini,[2] Matthew Broadbent, [1] Brice Darroch, [1] Olga A. Mass,[3] Alexei Nabok,[4] P. Leslie Dutton,[2] C. Neil Hunter,[5] Päivi Törmä[6] and Graham J. Leggett[1]

[1]Department of Chemistry, University of Sheffield, Brook Hill, Sheffield S3 7HF, UK; [2]The Johnson Research Foundation and Department of Biochemistry and Biophysics, University of Pennsylvania, Philadelphia, PA 10104, USA; [3]N. Carolina State University, Department of Chemistry, Raleigh, NC 27695 USA; [4]Materials and Engineering Research Institute, Sheffield Hallam University, Howard St, Sheffield S1 1WB, UK; [5]Department of Molecular Biology and Biotechnology, University of Sheffield, Western Bank, Sheffield S10 2TN, UK; [6]COMP Centre of Excellence, Department of Applied Physics, Aalto University, School of Science, P.O. Box 15100, 00076 Aalto, Finland

Materials and Chemicals

Microscope coverslip slides (22 mm x 50 mm, #1.5 thickness) were obtained from Menzel-Gläser, Germany. Gold wire (99.997% trace metals basis) and chromium chips (99.5% trace metals basis) used for the thermal evaporation were purchased from Sigma-Aldrich. The 30% hydrogen peroxide solution and 95% concentrated sulfuric acid used for preparation of the piranha solution were supplied by VWR Chemicals, UK. For preparation of the gold etchant solution, 32% ammonia solution, HPLC purity ethanol, and cysteamine, obtained from Sigma-Aldrich, were used. 1-Octadecanethiol (98%), and HEPES were also obtained from Sigma-Aldrich. Chemicals used for the gold nanostructures functionalization, ie. 11-amino-1-undecanethiol hydrochloride, glutaraldehyde (25%), *N*α,*N*α-bis(carboxymethyl)-L-lysine trifluoroacetate salt (AB-NTA), and nickel sulfate were supplied by Sigma-Aldrich. All chemicals were used as received.

Fabrication of Gold Nanostructures

All glassware, i.e. the microscope coverslip slides and vials used were cleaned initially by submersion in piranha solution, a mixture of hydrogen peroxide and concentrated sulfuric acid in the ratio 3:7, for 40 – 60 min, until the solution has stopped bubbling and cooled down to room temperature. The glassware was rinsed thoroughly with deionized water and sonicated for 10 min before being placed in the oven (ca. 90 ºC) to dry.

Gold substrates were prepared by evaporating a 3-5 nm thick chromium film followed by a 20 - 22 nm (unless otherwise is stated) thick gold layer. Chromium and gold were both deposited by thermal evaporation using an Edwards Auto 306 bell jar vacuum coating system under pressure of 8×10^{-7} mbar. Evaporation rates of 0.1 nm s^{-1} for Cr and 0.1 – 0.2 nm s^{-1} for Au were used. It should be noted, that the above-stated thickness values were taken from the evaporator QCM thickness monitor. They may differ (up to 8%) from the actual thickness values, which were determined later on by spectroscopic ellipsometry.

Chromium/gold coated glass slides were immersed in 1 mM solution of 1-octadecanethiol (ODT) in ethanol for at least 24 h to form closely packed self-assembled monolayers (SAMs). SAMs of ODT on gold were photopatterned by interferometric lithography (IL) using a Lloyd's mirror two-beam interferometer in conjunction with the frequency-doubled argon ion laser emitting at 244 nm (Innova FredD 300C, Coherent, UK). The angle between the mirror and the sample in the interferometer was 30 ± 2.5º. Samples were patterned using the IL with a dose 34 J cm^{-2}. Subsequently, samples were rotated by different angles on the sample stage and exposed again, to a dose of 20 J cm^{-2}.[1]

Photopatterned ODT monolayers on gold were etched by immersion in 2 mM cysteamine with an added 8% v/v of ammonia in HPLC ethanol. After etching, the samples were then rinsed with ethanol, dried under a steam of nitrogen and annealed in a chamber furnace (Carbolite, UK) at 500-550 ºC for 60 – 90 min. The heating rate was ca. 7 °C min^{-1} and the annealed samples were left to cool in air to room temperature. Highly crystalline structures and strong plasmon bands were observed in extinction spectra after annealing.

Samples were cleaned for re-use by immersion in piranha solution (which was allowed to cool down to room temperature) for 5 – 7 min, washed thoroughly with deionized water and blown dry with nitrogen.

Maquettes containing the SE369 chlorin[2] were over-expressed in BL-21DE3 strain *Escherichia coli* cells and purified as described previously,[3, 4] buffer exchanged into 20mM CHES, 150mM KCl, pH 9.0 buffer. Synthetic chlorin stock solution of final concentration 1mM in dimethyl sulfoxide (DMSO) was prepared, two and one equivalents of this stock solution were added drop-wise to the double and single Histidine maquette solutions respectively by gently stirring and incubating in the dark at room temperature for one hour. This chlorin bound maquette solution is purified through a PD-10 size exclusion column (GE healthcare) to remove DMSO, and any free chlorin.

Surface Functionalization and Maquette Attachment

Arrays of gold nanostructures were functionalized with 11-amino-1-undecanethiol (AUT) by immersion in a 2 mMol solution of the adsorbate in ethanol for 18 h, washed with ethanol and dried with nitrogen/ The SAMs were derivatised by reaction with glutaraldehyde (12.5% in water) for 40 min, and then immersed in a 10 mM aqueous solution of *N*α,*N*α-bis(carboxymethyl)-L-lysine trifluoroacetate salt, pH 5 for at least for 2 h. Them the samples were rinsed with deionized water, dried under a steam of nitrogen and immersed in a 10 mM aqueous solution of nickel sulfate for 5 min to ensure complexation of NTA by Ni^{2+}. The samples were washed thoroughly with deionized water and dry under a stream of nitrogen to remove excess Ni^{2+}. The samples were then immersed in His-tagged BT6 Maquette in 20 mM HEPES buffer, 0.03% BDDM, pH 7.5. The surfaces were immersed in the maquette/buffer solution for overnight in a humid chamber in a fridge. Finally, the samples were then gently washed with HEPES buffer and deionized water and dried under a steam of nitrogen.

Characterization

Atomic Force Microscopy (AFM). Morphology of the as-fabricated and annealed gold nanostructures was determined with AFM. AFM images were acquired in air using a Nanoscope Multimode 8 atomic force microscope (Bruker, Germany) operated in a tapping mode. Tapping mode probes used were OTESPA-R3 model (Bruker), with a resonance frequency of ca. 300 kHz and a nominal tip radius of 7 nm. Image analysis was performed with the Bruker NanoScope Analysis (v.1.5) software.

X-ray photoelectron spectroscopy (XPS). To characterize the reaction sequence in Figure 1b in the main text, XPS measurements were performed using a Kratos Axis Ultra X-ray photoelectron spectrometer (Kratos Analytical, Japan), equipped with a delay-line detector. The operating pressure was 1×10^{-9} mbar. Survey spectra were acquired at pass energy of 160 eV and high-resolution spectra were acquired at pass energy of 20 eV. All XPS spectra were analyzed and curve-fitted using the Casa XPS software (v.2.3.15) and were corrected relative to the C 1s signal at binding energy (B.E.) = 285.0 eV. Measurements were repeated three times, and errors quoted in numerical data are the standard deviations.

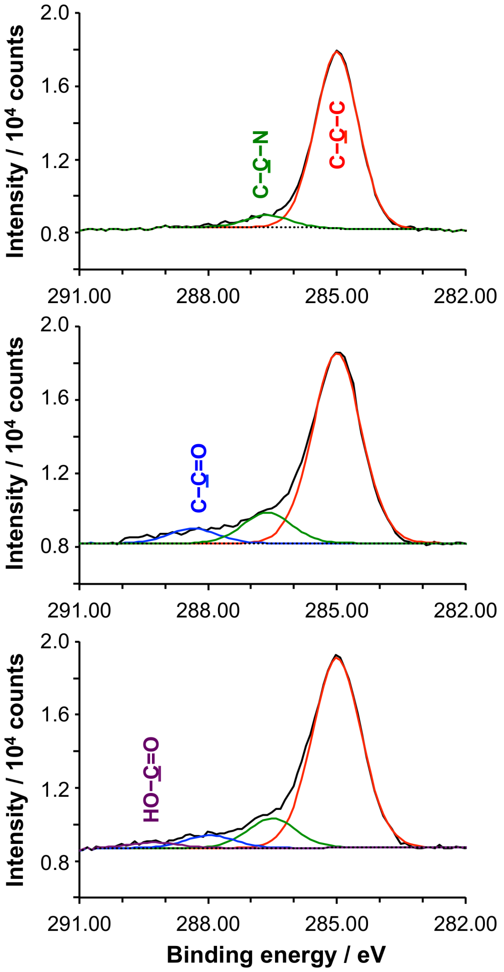

Figure S1. XPS C1s spectra of (a) an AUT SAM; (b) an AUT SAM after reaction with glutaraldehyde; (c) an NTA-functionalised monolayer. See main text for discussion of the significance of these spectra.

After reaction of glutaraldehyde with an AUT SAM, a component was observed at 288.4 eV corresponding to the aldehyde terminal group. The area of this peak was 5.6 at %, compared to a calculated value of 12.5 at % assuming complete reaction, indicating slightly less than 50% derivatization of the surface, consistent with previous studies on aminosilane systems by Xia et al.[5] After the final step in the surface derivatization process, attachment of aminobutyl NTA, a carboxylate peak is observed at 289.0 eV, with an area of 1.8 at %, compared to a calculated value of 5.6 at %. Although the overall yield is 32%, it needs to be borne in mind that surface reactions often have poor yields, because of the severe steric constraints, and moreover, this process involves multiple steps. Given that the cross-sectional area of a BT6 maquette is ~4 nm^2, and the efficiency of NTA-His complexation is high, this density of NTA groups should be adequate for the formation of a close-packed layer of maquettes.

Spectroscopic ellipsometry. The variable angle spectroscopic ellipsometer M-2000V (J. A. Woollam, USA) was used to determine the efficiency of the maquette attachment method, also to reveal the thicknesses of adsorbed layers on continuous gold film. The nominal thickness of the evaporated chromium/gold layers were 3 nm and 35 nm, respectively. Angle of incidence was fixed at 70° and the spectral range was 370 – 1000 nm. Ellipsometric data were acquired in air and analyzed using the CompleteEASE (v.4.92) software. Fitting of the experimental spectra $\Psi(\lambda)$ and $\Delta(\lambda)$ was carried out using the model presented in the Table S1. A Cauchy dispersion function $n = A_n + \frac{B_n}{\lambda^2} + \frac{C_n}{\lambda^4}$, (preferable for determining the optical

constants of a transparent or partially transparent film) was used for fitting the adsorbed layer. The obtained thicknesses of chromium, gold, and Cauchy layers, together with mean squared error (MSE), are summarized in Table 1 (main text).

Table S1. The model used for spectroscopic ellipsometry data fitting.

Layer	***Material***	***Fitted parameters***	***comments***
Layer #3	Cauchy	*d, A*	*d* is the thickness of the adsorbed layer in nm. The parameters B_n=0.01 and C_n=0 were fixed during the fitting.
Layer #2	Au	*d*	thickness of the evaporated gold film in nm
Layer #1	Cr	*d*	thickness of the evaporated chromium film in nm
Substrate	BK7 glass	-	*n* = 1.515

UV-vis spectroscopy. UV-visible absorption spectra at normal incidence were recorded in air using a Cary50 spectrophotometer (Agilent Technologies, USA). The wavelength scan range was 350–850 nm (unless otherwise stated). The samples were placed in a special holder enabling absorption measurements of the same spot on the sample during all experimental stages. The concentration of maquette was determined by measuring the absorption at λ=415 nm (ε=3500 $mM^{-1}cm^{-1}$).

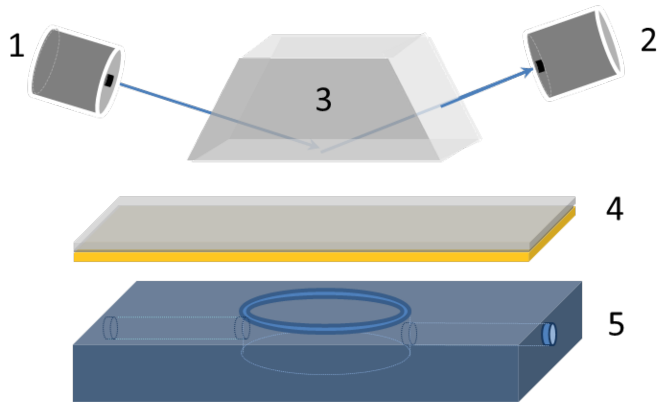

Figure S2. Schematic diagram showing the cell used for TIRE measurements. The cell is based on rotating compensator spectroscopic ellipsometer. 1 – polarizer, 2 – analyzer, 3 – 68° prism, 4 – C/Au coated glass slide (attached to the bottom of the prism via index-matching liquid), 5 – cell with inlet and outlet tubes.

Total Internal Reflection Ellipsometry (TIRE). The kinetics of attachment of maquettes on to NTA/Ni^{2+}-functionalized gold surfaces was studied using the method of total internal reflection ellipsometry (TIRE).[6] The TIRE method joins the spectroscopic ellipsometry and the Kretschmann configuration surface plasmon resonance (SPR). The TIRE measurements were performed on the rotating compensator spectroscopic ellipsometer M-2000V (J. A. Woollam, USA) *in situ* in a Teflon cell of 0.2 ml in volume, attached to the bottom of a 68° trapezoidal glass prism via Cr/Au coated coverslip glass slide, as shown in the Figure S2. The angle of incidence was set at 68°. The wavelength scan range was 370–1000 nm. The TIRE data were acquired and analyzed using the ComleteEASE software program.

The nominal thicknesses of the unpatented, polycrystalline chromium/gold films, used for these measurements, were 11 nm and 50 nm, respectively. The 68° prism coupled the light

beam into thin gold film at the conditions close to total internal reflection. In contrast to the conventional SPR, based on measuring of intensity of reflected p-polarized light, the method of TIRE detects two ellipsometric parameters: Ψ and Δ. The parameter Ψ is related to the amplitude ratio as $tn\Psi = \frac{A_p}{A_s}$ and is similar to the SPR curve. The parameter $\Delta = \varphi_p - \varphi_s$ is the phase shift between p- and s- components of polarized light.

In TIRE measurements, the adsorption of the maquette on NTA/Ni^{2+}-functionalized gold films was carried out in in the same aqueous buffer environment. $\Psi(\lambda)$ and $\Delta(\lambda)$ spectra were recorded at the starting moment of the adsorption of maquette as well as at the end of varying periods of time with an intermediate rinse with buffer in order to remove the excess maquette. Figure S3 shows a typical set of $\Psi(\lambda)$ and $\Delta(\lambda)$ TIRE spectra in the HEPES buffer just before injection of maquette in the cell and after 80 min of adsorption of maquette.

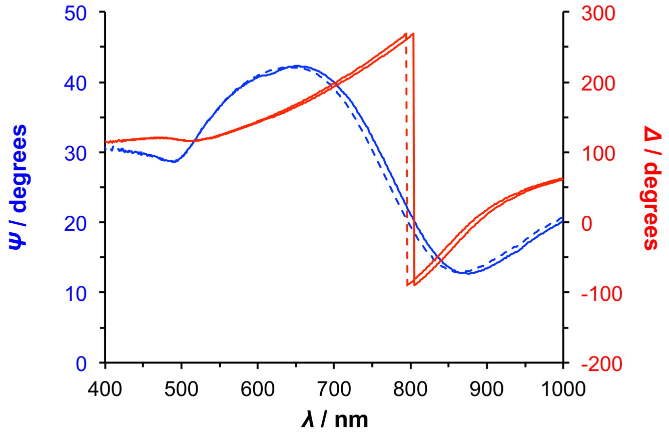

Figure S3. A typical set of $\Psi(\lambda)$ and $\Delta(\lambda)$ TIRE spectra measured in the HEPES buffer before (dashed lines) and after (solid lines) 80 min of adsorption of maquette on NTA/Ni^{2+}-functionalized gold film.

Table S2. The model exploited for TIRE data fitting.

Layer	***Material***	***Fitted parameters***	***comments***
Layer #4	Cauchy	*A, d*	Parameters B = 0.01 and C = 0 were fixed during the fitting.
Layer #3	Cauchy	-	thickness of the (11-amino-1-undecanethiol/glutaraldehyde/NTA/Ni^{2+}) layer was determined separately (d = 1.52 ± 0.04 nm) and was fixed during the fitting
Layer #2	Au	*d*	thickness of the evaporated gold film in nm
Layer #1	Cr	*d*	thickness of the evaporated chromium film in nm
Substrate	BK7 glass	-	n = 1.515
Ambient	Water		

These measurements were repeated for the three concentrations of maquette, namely, 511 nM, 83 nM, and 31 nM. The experimental spectra $\Psi(\lambda)$ and $\Delta(\lambda)$ were fitted to the model presented in the Table S2 using the CompleteEASE software.

The kinetics of adsorption of maquettes onto NTA/Ni^{2+}-functionalized unpatterned, polycrystalline gold films was studied by measuring TIRE spectra every 0.15 s during the varying periods of time. Then time dependences of either Ψ or Δ can be obtained at the spectral range 370–1000 nm. The time dependence of Ψ was used, since $\Psi(\lambda)$ spectra give a wider linear range as compared to $\Delta(\lambda)$ spectra. A wavelength of 750 nm was selected at the linear section of $\Psi(\lambda)$ spectrum (see Figure S3). These measurements were repeated for three different concentrations of maquettes for a period of time varied from 2 to 160 min. A typical set of $\Psi(t)$ and $\Delta(t)$ spectra is presented in Figure S4.

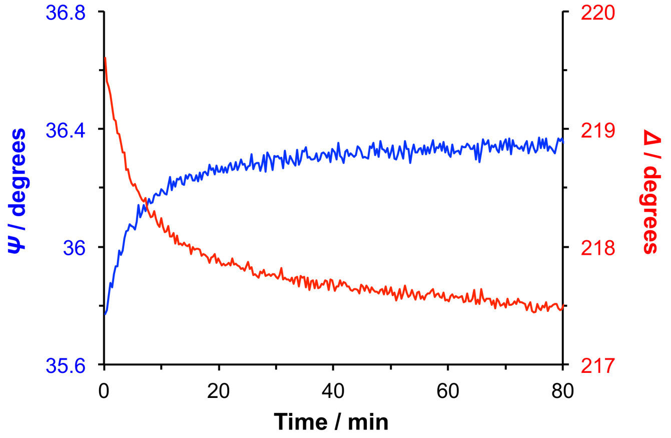

Figure S4. A typical set of $\Psi(t)$ and $\Delta(t)$ TIRE spectra measured during the immersion of NTA/Ni^{2+}-functionalized polycrystalline gold films in a 511 nM solution of BT6-SE369 maquette in HEPES buffer.

References

1. S. R. J. Brueck, *Proc. IEEE*, 2005, **93**, 1704-1721.
2. K. Aravindu, O. Mass, P. Vairaprakash, J. W. Springer, E. Yang, D. M. Niedzwiedzki, C. Kirmaier, D. F. Bocian, D. Holten and J. S. Lindsey, *Chem. Sci.*, 2013, **4**, 3459-3477.
3. T. A. Farid, G. Kodali, L. A. Solomon, B. R. Lichtenstein, M. M. Sheehan, B. A. Fry, C. Bialas, N. M. Ennist, J. A. Siedlecki, Z. Zhao, M. A. Stetz, K. G. Valentine, J. L. R. Anderson, A. J. Wand, B. M. Discher, C. C. Moser and P. L. Dutton, *Nat. Chem. Biol*, 2013, **9**, 826-833.
4. L. A. Solomon, G. Kodali, C. C. Moser and P. L. Dutton, *J. Am. Chem. Soc.*, 2014, **136**, 3192-3199.
5. S. Xia, M. Cartron, J. Morby, D. A. Bryant, C. N. Hunter and G. J. Leggett, *Langmuir*, 2016, **32**, 1818-1827.
6. M. Poksinski and H. Arwin, *Thin Solid Films*, 2004, **455-456**, 716-721.
